# A hybrid neighborhood enhanced contrastive learning and self-knowledge distillation method for scRNA-seq data clustering analysis

**DOI:** 10.1093/bioinformatics/btag084

**Published:** 2026-03-29

**Authors:** Lihua Qi, Peng Wang, Hao Liu, Chen Chen, Jin Gu, Cheng Chen

**Affiliations:** School of Computer Science and Technology, Xinjiang University, Urumqi, 830046, China; Department of Dermatology and Venereology, People’s Hospital of Xinjiang Uygur Autonomous Region, Urumqi, Xinjiang 830000, China; Xinjiang Clinical Research Center for Dermatologic Diseases, Urumqi, Xinjiang 830000, China; Xinjiang Key Laboratory of Dermatology Research (XJYS1707), Urumqi, Xinjiang 830000, China; School of Computer Science and Technology, Xinjiang University, Urumqi, 830046, China; School of Software, Xinjiang University, Urumqi, 830046, China; Key Laboratory of signal detection and processing, Xinjiang University, Urumqi, 830046, China; Xinjiang Cloud Computing Application Laboratory, Karamay, 834099, China; MOE Key Laboratory of Bioinformatics, BNRIST Bioinformatics Division, Institute for Precision Medicine & Department of Automation, Tsinghua University, Beijing, 100084, China; School of Software, Xinjiang University, Urumqi, 830046, China; Xinjiang Key Laboratory of Cardiovascular Homeostasis and Regeneration Research, Urumqi, 830000, China

## Abstract

**Motivation:**

Single-cell heterogeneity analysis faces significant challenges due to the high dimensionality, complexity, and noise inherent in scRNA-seq data, especially when aiming for precise cell type classification. Existing analytical methods often exhibit limited generalization ability and adaptability across different biological contexts, leading to biased identification of cell subpopulations and hindering a comprehensive understanding of diseases, therapeutic responses, and biological processes.

**Results:**

To address these issues, we propose a novel method named scKD, which integrates a hybrid neighbourhood-enhanced comparative learning model with a self-knowledge distillation strategy. scKD enhances clustering accuracy and is capable of accurately identifying both major cell types and rare cell subtypes. Extensive evaluations on multiple real-world datasets demonstrate that scKD achieves superior performance in subpopulation identification, clustering stability, and robustness. These results suggest that scKD is a powerful and reliable tool for analyzing single-cell transcriptomic data, facilitating deeper insights into cellular heterogeneity.

**Availability:**

All datasets used in this study are publicly available. Detailed information about all the single-cell datasets analyzed in this paper is provided in Supplementary Table 1. All datasets can be accessed at https://zenodo.org/records/15412380. The source code is available at https://github.com/A-qlh/sckd.

**Supplementary information:**

Supplementary data are available at Bioinformatics online.

## 1 Introduction

Rapid advances in single-cell transcriptomics (scRNA-seq) have propelled the study of tissue heterogeneity into a whole new era, enabling insights at the cellular level (Kharchenko 2021). This technique can accurately quantify the transcriptome of individual cells and reveal the genetic and functional diversity of cells, which provides important support for in-depth study of intricate biological mechanisms like cell differentiation and gene regulation, and provides a valuable tool for resolving heterogeneity among cells. Previously, the traditional bulk RNA-seq technique investigated gene expression by analysing mixed samples from a large number of cells, an approach that focuses mainly on revealing the average transcript levels of a population of cells, but fails to differentiate between gene expression differences between individual cells and may mask rare cell populations, minor transcriptional differences, or differences in gene expression over time (Sun *et al.* 2019). scRNA-seq technology has emerged to overcome these limitations by accurately identifying both predominant cell types and infrequent cell types, offering a fresh perspective in the study of cellular heterogeneity. This technique allows for a deeper understanding of intercellular diversity, thereby uncovering the complexity and dynamics within cell populations at the individual cell scale (Chen *et al.* 2021).

In conventional scRNA-seq data analysis workflows, certain approaches utilize methods like principal component analysis (PCA) and the K-means clustering algorithm for reducing dimensionality and performing cluster analysis (Luecken and Theis 2019). However, these methods have limitations, PCA, being a linear technique for dimensionality reduction, struggles to capture complex nonlinear structures within the data, and its effectiveness is notably reduced when handling noisy scRNA-seq data (Townes *et al.* 2019). In addition, the K-means clustering technique is particularly susceptible to outliers, which may result in unstable clustering outcomes and difficulty in identifying the global optimum (Dana *et al.* 2021).

Despite the strong intuition and high ease of use of these traditional methods, they still face certain challenges in terms of performance and adaptability. For instance, PCA struggles to capture non-linear relationships and handle noise, whilst K-means is sensitive to outliers, leading to unstable results and failing to guarantee global optimality. As such, a growing number of studies have begun to explore the application of deep learning methods to improve and optimize the representation learning process for scRNA-seq data, aiming to enhance its accuracy and effectiveness. As a feature learning approach, deep learning does not merely optimize the clustering process, but rather enhances the entire data analysis pipeline—including clustering—by extracting deeper features that capture nonlinear patterns, latent structures, and complex dependencies in high-dimensional spaces (Lin *et al.* 2022). This approach better captures the complex structures and relationships within the data, thereby improving the precision and biological relevance of clustering. For example, scANVI is a semi-supervised deep learning method based on variational inference, which integrates prior cell type annotations to achieve joint representation learning and cell type annotation of scRNA-seq data, while also demonstrating strong performance in data integration and batch effect correction (Xu *et al.* 2021). In addition, scAnCluster fuses deep supervised learning, self-supervised learning, and unsupervised strategies to harness the cell type annotations from reference datasets, assisting in the clustering and annotation of unlabeled datasets (Chen *et al.* 2020c). However, although scAnCluster accounts for batch effects to some extent, additional batch correction steps may be required in the presence of strong batch effects. scZidesk model takes into account the limitations of scAnCluster by using a deep denoising self-encoder, which is well-suited for better characterising scRNA-seq data and removing, in the downscaling process, the noise, thus obtaining a clearer structure of the cell population (Chen *et al.* 2020a). At the same time, GapClust, an emerging clustering algorithm, demonstrates notable advantages in processing extensive scRNA-seq datasets. The algorithm efficiently identifies rare cell subtypes within complex scRNA-seq data by utilizing the variations in distance between data points (Fa *et al.* 2021). The scSID algorithm is also a streamlined method developed to detect infrequent cell types. It identifies these cell types by analyzing differential expression in the scRNA-seq data, considering both intercellular similarities and the variation in similarities within and between cell clusters (Wang *et al.* 2024). Building on these advances in transcriptomic clustering, the application of deep learning in single-cell analysis has further expanded into spatial transcriptomics and more sophisticated self-supervised frameworks. For instance, the stAA framework proposed by Fang *et al.* (2024) significantly enhances spatial domain recognition accuracy in spatial transcriptomics data by integrating an adversarial variational graph autoencoder with graph neural networks (GNNs) and Wasserstein generative adversarial networks (WGANs). This approach demonstrates the potential of deep learning in integrating spatial information with gene expression data, offering novel insights for processing high-dimensional, complex single-cell data. Furthermore, the CAKE framework proposed by Liu *et al.* (2024) employs a self-knowledge distillation strategy to enhance clustering performance on scRNA-seq data. CAKE directly optimizes a low-dimensional embedding space using a momentum contrastive objective, in which different augmented views of the same cell and its k-nearest neighbours are treated as positive pairs. This design alleviates the class-collision problem and yields cluster-friendly representations. In the second stage, CAKE further refines cluster assignments by performing self-distillation on high-confidence cells. Notably, CAKE does not rely on an explicit count-based generative autoencoder, but instead focuses on learning discriminative embeddings for visualisation, clustering and rare cell type identification. Furthermore, the scCoRR framework proposed by Zheng *et al.* (2025) addresses the issue of coarse labelling in single-cell RNA sequencing data by developing a data-driven cell label correction method. scCoRR identifies reliable anchor cells through neighbourhood purity and global selection strategies, then optimises the prediction model by combining classification loss with contrastive regularisation to correct labels for remaining cells. This approach significantly enhances cell type annotation accuracy without requiring reference datasets or labelled genes, laying the groundwork for objective evaluation of subsequent clustering methods. These studies demonstrate that deep learning approaches possess distinct advantages in addressing the sparsity, high dimensionality, and noise inherent in single-cell RNA sequencing and spatial transcriptomics data. Concurrently, they provide novel tools for analysing cellular heterogeneity and achieving precise annotation.

Faced with the challenge of acquiring labelled data, self-supervised contrast learning techniques have shown their strong potential in the image and text domains, sometimes even surpassing traditional supervised learning strategies (Nadif and Role 2021). Therefore, applying the concept of contrast learning to scRNA-seq data analysis is an area worth exploring. However, there are challenges in directly applying contrastive learning methods, initially developed for images and text, to scRNA-seq datasets, as these approaches typically depend on the spatial structure or semantic information, whereas scRNA-seq data has distinct characteristics in these regards (Yoon *et al.* 2020).

Given that scRNA-seq data often exhibit sparsity and high dimensionality, directly applying standard contrastive learning methods to such data may face several challenges. Firstly, the sparsity and high-dimensional nature inherent in scRNA-seq data represent a major issue. Many genes are not expressed in individual cells, resulting in most of the data being zero-valued, which makes it difficult for standard contrastive learning methods to effectively capture potential features in the sparse matrix. In addition, noise and dropout events within the data (i.e., the failure to detect some gene expression signals) can also affect the model’s discrimination between similarities and differences between cells, which in turn leads to erroneous clustering or representation learning. Therefore, to tackle these issues, specific adjustments and optimizations of these methods are required to better match them with the unique features of scRNA-seq data (Hie *et al.* 2020). In the case of scNAME, for example, the method employs contrastive learning to reveal the intrinsic cellular structure by combining the tasks of gene correlation mining and mask estimation. The patterns learned from mask estimation not only effectively capture the structure of data unaffected by noise but also reduce noise, thereby enhancing the precision and consistency of clustering (Wan *et al.* 2022). In addition, CLEAR introduces a novel scRNA-seq data contrastive learning method, which generates positive sample pairs (altered expression profiles of identical cells) and negative sample pairs (expression profiles from different cells) by simulating technical noise and dropout events through data augmentation methods. During training, CLEAR learns an effective representation of scRNA-seq data by employing a contrastive loss function that prompts the model to produce similar low-dimensional representations for positive sample pairs and dissimilar representations for negative sample pairs (Han *et al.* 2022). These studies demonstrate that contrastive learning can be successfully adapted to scRNA-seq data, but also reveal remaining limitations in neighbourhood modelling, noise robustness and clustering‑oriented optimisation.

Considering the limitations and strengths of previously proposed scRNA-seq analysis methods, this study presents a new deep learning framework, termed scKD, for robust single-cell clustering. The primary objective of scKD is to enhance the accuracy and robustness of cell clustering by integrating a hybrid neighbourhood‑enhanced contrastive learning module with a clustering‑oriented self‑knowledge distillation strategy, thereby enabling deeper exploration of intercellular similarity structures and feature correlations. In contrast to contrastive learning–based methods such as scNAME and CLEAR, which mainly treat different augmented views of the same cell as positive pairs, scKD explicitly incorporates reliable neighbours into the positive set and jointly optimises denoising, ZINB‑based count modelling and neighbourhood‑aware contrastive learning within a unified autoencoder framework. Compared with distillation‑based approaches such as CAKE that focus on knowledge transfer for cell type annotation, scKD adopts a self‑distillation scheme tailored for unsupervised clustering: high‑density anchor cells in the learned representation space provide both hard labels and soft pseudo‑labels to iteratively refine ambiguous or boundary cells, targeting improved clustering quality rather than generic model compression or supervised classification.

Concretely, scKD first learns enhanced cell representations through the hybrid neighbourhood‑enhanced contrastive learning module. The preprocessed gene expression matrix is then projected by the trained contrastive encoder to obtain a subset of high‑confidence cells for subsequent training and evaluation. On this basis, a self‑knowledge distillation strategy is employed, where the contrastive‑pretrained encoder acts as a teacher to guide a student network using both hard cluster assignments and softened probability distributions, ultimately yielding more fine‑grained and reliable clustering labels. Experiments conducted on multiple real scRNA‑seq datasets demonstrate that scKD consistently surpasses competing contrastive learning and knowledge distillation–based models, including scNAME and CLEAR, in terms of clustering accuracy, robustness and scalability.

## 2 Methods

### 2.1 Datasets

To comprehensively assess the performance of scKD for scRNA-seq data analysis, experiments were conducted using seven distinct, authentic, and well-annotated scRNA-seq datasets obtained from recent studies. The datasets used in this study cover a wide range of cell types and biological processes across different species and tissues, including the human pancreas, and mouse spleen, providing a comprehensive basis for the analyses conducted. In addition, the datasets were generated using various sequencing technologies, including Smart-seq2, 10x Genomic, inDrop, and CELseq2. e.g., the Muraro *et al.* (2016) dataset originated from the human pancreas, using CEL-seq2 sequencing technology. The datasets were derived from mouse hearts and sequenced using Smart-seq2. Before inputting the data into the scKD model, each dataset underwent standard preprocessing procedures to ensure data quality and consistency. These steps included filtering out genes expressed in fewer than three cells, normalization of gene expression to account for sequencing depth, and log-transformation to stabilize variance. Subsequently, the 2000 most variable genes were selected for downstream analysis, as they are critical for accurate cell type classification. Details of all datasets are provided in Supplementary Table 1. To evaluate the robustness of highly variable gene selection, we tested the impact of using 1,000 variable genes on the experimental results, as shown in Supplementary Table 2.

### 2.2 General framework of the scKD

**Figure 1:**
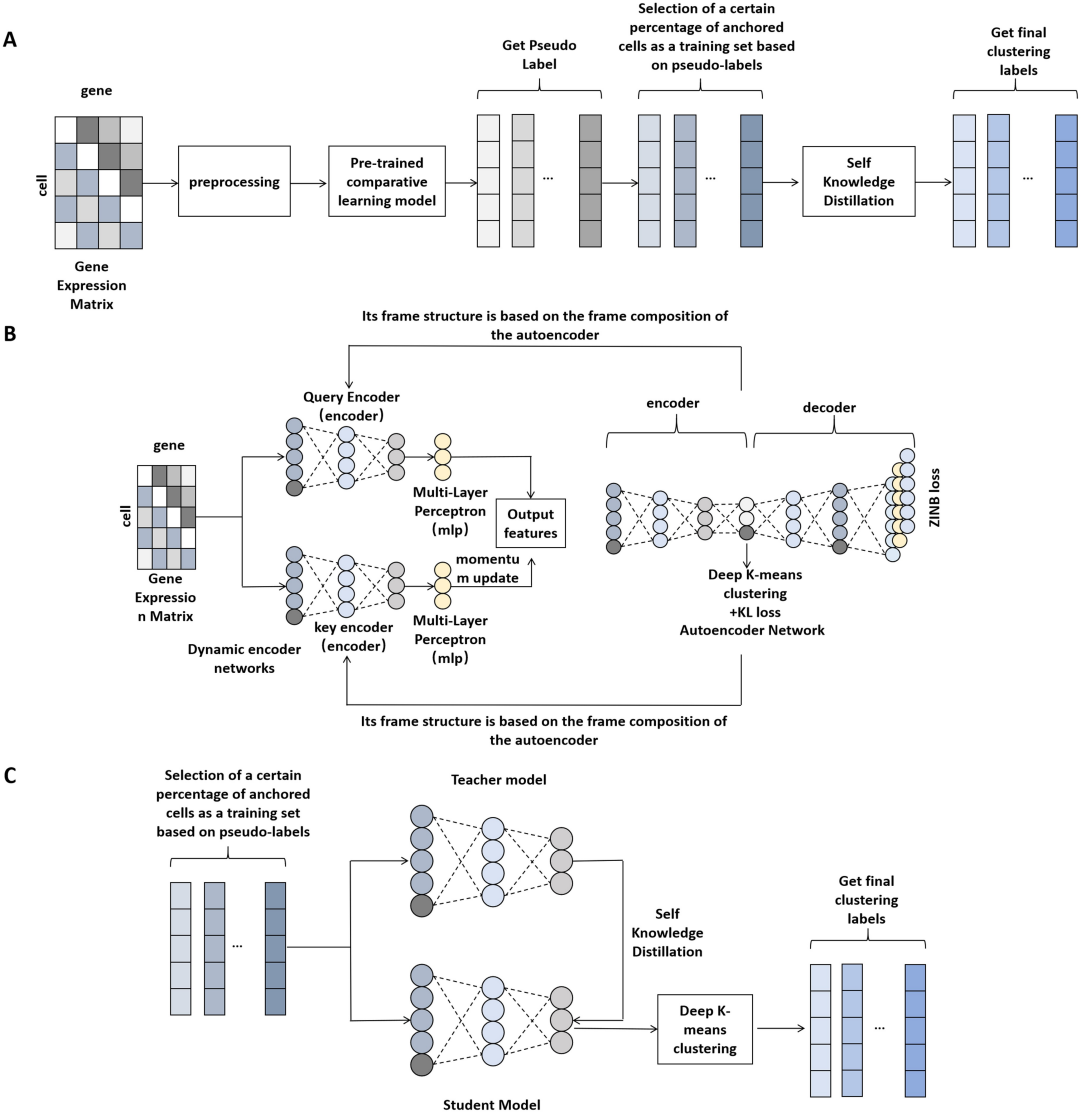
(A) Process framework diagram of sczKD; (B) Schematic diagram of hybrid neighbourhood-enhanced contrast learning network for sczKD. The basic framework of the contrast learning network is built with a denoising ZINB model-based self-encoder framework. The encoder and decoder parts of this self-encoder framework present a symmetric layout, and in the low-dimensional bottleneck layer, the embedding points are clustered using a weighted soft K-mean clustering algorithm with a self-training process. The purpose of the adaptive training strategy is to pool cell samples with similar features while working on finding a potential space more favourable for cluster analysis. (C) Schematic diagram of the self-knowledge distillation network. Self-knowledge distillation is a special form of knowledge distillation in which the student model and the teacher model are the same model. In this study, the teacher model is built based on the encoder framework of the contrastive learning model, which has learnt some useful representations of the data in the pre-training phase.

The scKD model implements a two-phase self-supervised learning approach to analyze gene expression data, as illustrated in Fig. 1(A). firstly, a self-encoder model is used to construct the network structure base of the model (for more information, see Section 2.4). Inspired by the contrastive learning model in scNAME, this study constructed a dynamic encoder network for enhanced contrastive learning, including a query encoder and a key encoder (for more information, please refer to Section 2.5), as shown in Fig. 1(B), whose primary goal is to generate feature representations for the contrastive learning task and enhance the stability and performance of the system through a dynamic updating mechanism, thereby boosting the efficacy of contrastive learning. A multilayer perceptron (MLP) header is added after the output of the model’s encoder for further processing of the features, with the aim of mapping the features learnt by the encoder into a space more conducive to contrast learning, thus allowing the system to acquire high-quality data representations more effectively (Singh and Singh 2020). The pre-processed gene expression data were predicted using the trained model, while the Leiden algorithm was used to assign identifiers to each cell for initial clustering based on the learned representations. Using these identifiers, a refined set of high-quality data was constructed, which consisted of a high density of anchored cells from each clustering (for more information, please refer to Section 2.6). Next, in order to further optimise the clustering results, a self-knowledge distillation (KD) strategy is applied to perform knowledge distillation (Baron *et al.* 2016) on the data subset obtained in the earlier step, as depicted in Fig. 1(C), thereby refining the clustering outcomes and ultimately assigning probabilistic and detailed clustering identifiers to the cells.

### 2.3 Denoising Self-Encoder based on ZINB model

A self-encoder is a model that efficiently learns a linear mapping of the data and extracts meaningful feature representations through an unsupervised learning approach (Wang *et al.* 2024) Denoising self-encoders are widely applied to learn robust latent representations of noisy datasets by processing data corrupted with artificial noise and reconstructing the untainted original version (Lipkova *et al.* 2022). As a particular form of self-encoder, denoising self-encoders take in degraded data as input and are trained to reconstruct the original, undistorted data as output (Fang *et al.* 2024). This kind of self-encoder typically includes a narrow bottleneck layer designed to capture the essential feature representations of the data. Due to the high dimensionality and sparsity of scRNA-seq data, these characteristics pose significant challenges in scRNA-seq data analysis. Therefore, denoising self-encoders were employed to process the scRNA-seq data in this study (Hie *et al.* 2020). The denoising self-encoder was used to map gene expression matrices and convert them into low-dimensional embeddings for enhanced efficiency in analyzing scRNA-seq data.

In terms of implementation, a self-encoder is built using standard fully-connected layers, where the input is first corrupted by random Gaussian noise.

Specifically, the input X is altered by a Gaussian noise e, represented as $X^{\mathrm{corrput}}=X+e$. It is important to highlight that noise can be integrated into the layers of the self-encoder, a technique referred to as cascaded denoising self-encoder (Sahay *et al.* 2019). The encoder function is denoted as $z=f_{w}(X^{\mathrm{corrput}})$ and the decoder function as $X^{'}=g_{w^{'}}(z)$, where W and W' represent the learning weights of the encoder and decoder, respectively. Additionally, during the training of the denoising self-encoder, the objective loss function is $L(X,g_{w^{'}}(f_{w}(X^{\mathrm{corrput}})))$, which measures the discrepancy between predicted and actual values. To accurately capture the underlying features of scRNA-seq data, this study utilizes a model based on the Zero-Inflated Negative Binomial (ZINB) distribution as the reconstruction loss, which replaces the traditional self-encoder with one designed for zero-inflated negative binomial data (Singh and Singh 2020). Unlike the conventional self-encoder, the loss function in this case is derived from the likelihood of the ZINB model. For implementation, the ZINB model is parameterized using the mean (μ), the dispersion parameter (θ) of the negative binomial distribution, and an additional coefficient (π) representing the probability of observing zero values, which corresponds to dropout events.


(1) $${\mathrm{NB}(X}^{\mathrm{count}}|\mu ,\theta )=\frac{\Gamma (X^{\mathrm{count}}+\theta )}{X^{\mathrm{count}}!\Gamma (\theta )}{\left(\frac{\theta }{\theta +\mu }\right)}^{\theta }{\left(\frac{\mu }{\theta +\mu }\right)}^{X^{\mathrm{count}}}$$

(2) $$\mathrm{ZINB}(X^{\mathrm{count}}|\pi ,\mu ,\theta )=\pi \delta_{0}(X^{\mathrm{count}})+(1-\pi )NB{(X}^{\mathrm{count}}|\mu ,\theta )$$

Where $X^{\mathrm{count}}$ denotes the original read count. μ, θ and π are the estimated parameters of the ZINB-based self-encoder.

Unsupervised clustering is applied to the latent space of a self-encoder utilizing the Zero-Inflated Negative Binomial (ZINB) model (Fang *et al.* 2024), where a nonlinear transformation is learned for each cell i, mapping the input matrix into a reduced-dimensional space Z. Deep K-means clustering is applied during the clustering phase. This step involves grouping n cells, denoted as X, where each cell $x_{i}\epsilon N^{d}$ (represents the i-th cell with d read count of a gene) is assigned to one of k clusters. Deep embedding clustering begins by discovering a nonlinear mapping $f_{w}∼x_{i}\to z_{i}$, where Z represents the latent feature space, typically much smaller than X.Clustering is then performed in the latent space Z. To extract the features of scRNA-seg data, a self-encoder based on the denoising ZINB model is utilized to learn the nonlinear transformation from X to Z.In scRNA-seg data analysis, the goal is to assign similar points to the same cluster. Following the approach outlined in the literature (Tian *et al.* 2019), the KL scatter loss function is applied to improve the similarity between cells, and for implementation, the clustering loss can be formulated as:


(3) $$L_{c}=\mathrm{KL}(P||Q)=\sum_{i}\sum_{j}p_{\mathrm{ij}}l\mathrm{og}\frac{p_{\mathrm{ij}}}{q_{\mathrm{ij}}}$$

The expression above represents the Kullback-Leibler (KL) divergence between distributions P and Q, where Q is a soft-label distribution determined using the Student’s t-distribution. During model training, P functions as the target distribution, aiming to enhance the connections between highly similar cells while reducing the connections between less similar cells. This method is employed to quantify the difference between the two probability distributions. For each training batch, both P and Q are computed. Following the approach of t-SNE (Pareek and Jacob 2020), the t-distribution is utilized to quantify the pairwise similarity between cells i and j in the potential space of the ZINB-based self-encoder. Let $q_{\mathrm{ij}}$ be the soft label of the embedding represent the soft label of the embedding point $z_{i}$, which is defined as the similarity between $z_{i}$ and the cluster center $\mu_{i}$ measured using the Student’s t-distribution and expressed as:


(4) $$q_{\mathrm{ij}}=\frac{{\left(1+{\left||z_{i}-\mu_{j}|\right|}^{2}\right)}^{-1}}{\sum_{j^{\prime }}{\left(1+{\left||z_{i}-\mu_{j}|\right|}^{2}\right)}^{-1}}$$

Let $p_{\mathrm{ij}}$ be the target distribution, we first lift $q_{\mathrm{ij}}$ to quadratic and then normalise by the frequency of each cluster, denoted:


(5) $$p_{\mathrm{ij}}=\frac{{q_{\mathrm{ij}}}^{2}/\sum_{j}q_{\mathrm{ij}}}{\sum_{j^{\prime }}\left({q_{ij^{\prime }}}^{2}/\sum_{j^{\prime }}q_{ij^{\prime }}\right)}$$

The training process can be considered a self-training procedure, as the target distribution P is derived from Q. Prior to the clustering phase, the self-encoder of the cascaded denoising ZINB model (Sahay *et al.* 2019) is pre-trained. The initialization of the clustering centers relies on the pre-trained feature space, which is derived by applying the standard K-means clustering algorithm. As a result, the encoder of the scKD model is composed of two components: the self-encoder from the denoising ZINB model and the clustering module. The objective function for optimization is:


(6) $$L=L_{\mathrm{ZINB}}+\gamma L_{c}$$

Where $L_{\mathrm{ZINB}}$ and $L_{c}$ represent the ZINB loss and the clustering loss of the base self-encoder from the denoising ZINB model, respectively. The parameter γ > 0 is a coefficient that adjusts the relative contribution of these two losses.

### 2.4 Dynamic encoder network based on an enhanced neighborhood contrastive learning

Contrastive learning is a method for self-supervised or unsupervised representation learning, aimed at differentiating between positive and negative samples within a dataset by training a neural network (He *et al.* 2020). This operation of training the network using augmented samples has significant advantages. Specifically, by adding random noise to the data, augmented data can be generated, which in turn enhances the robustness of the method for analyzing scRNA-seq data (Ciortan and Defrance 2021). In this study, the model employs the InfoNCE (Information Noise Contrastive Estimation) loss along with a momentum-based update mechanism. The corresponding InfoNCE loss (Parulekar *et al.* 2023) can be expressed as:


(7) $$L_{i}=-\log\frac{\exp(\frac{f{\left(x_{i}\right)}^{T}f({x_{i}}^{+})}{\tau })}{\exp(\frac{f{\left(x_{i}\right)}^{T}f({x_{i}}^{+})}{\tau }+\sum_{m=1}^{M}\exp(\frac{f{\left(x_{i}\right)}^{T}f({x_{i}}^{-})}{\tau })}$$

Where $f(x_{i})$ denotes the representation of the original sample $x_{i}$ after L2 regularisation. ${x_{i}}^{+}$ represents the positive sample corresponding to $x_{i}$ and ${x_{j}}^{-}$ refers to the negative sample related to $x_{i}$. Positive samples ${x_{i}}^{+}$ are typically created by introducing random noise or applying data augmentation techniques to the original sample $x_{i}$, while negative samples ${x_{j}}^{-}$ are obtained from other samples and their augmented versions (Gao *et al.* 2021). This method is applied in contrastive learning to model both the similarities and differences among samples, assisting the model in learning to differentiate between the feature representations of various samples. τ is a temperature coefficient that regulates the emphasis on distinguishing smaller instances and the balance between positive and negative pairs. M denotes the total number of negative samples. Typically, the InfoNCE loss definition consists of two parts, an alignment term and a homogeneity term. The alignment term promotes the positive samples ${x_{i}}^{+}$ to be close together, while the uniformity term ensures that samples are evenly distributed on the unit hypersphere by distancing the negative samples ${x_{j}}^{-}$ (Wang and Isola 2020). However, the inclusion of the uniformity term may lead to conflicts during unsupervised learning. This occurs when some ${x_{j}}^{-}$ are incorrectly pushed farther away from what should belong to the same class, which will complicate the subsequent analysis of scRNA-seq data (Wang and Isola 2020, Huang *et al.* 2023). In the current study, scKD establishes the foundational framework of a dynamic encoder by incorporating a self-encoder framework based on a denoising ZINB model. Additionally, it utilizes a neighborhood comparison learning enhancement strategy. The core concept behind this method is that cells with similar gene expression profiles are likely to belong to the same cell type, and therefore, such cells can be regarded as potential ${x_{i}}^{+}$. However, this manipulation needs to be handled with care, as there is a potential problem with the contrast loss function, i.e., it may incorrectly assume that all cells except amplification pairs are from different types, i.e., each cell is viewed independently as a cluster. Moreover, employing basic KNN visualizations could erroneously classify cells from distinct categories as belonging to a single category. This issue might result in the formation of extensive intermixed groups, thereby compromising the effectiveness of clustering, particularly when identifying rare cell types (Huang *et al.* 2023).

On this basis, a hybrid neighbourhood comparison enhancement strategy is proposed, which aims to simultaneously exploit the interrelationships between the original samples and their neighbours. In terms of implementation, scKD first searches the K nearest neighbours (KNNs) (Wang *et al.* 2023) of each original sample $x_{i}$, denoted as $X_{C}=\{x_{i1},x_{i2},\ldots x_{\mathrm{ic}}\}$, by the HNSW (Hierarchical Navigable Small World) method. scKD then employs a contrast learning architecture called Dynamic Encoder to obtain cellular representations, with a hybrid InfoNCE loss defined as follows.


(8) $${L_{i}}^{\mathrm{mixed}}=-\log\frac{\exp(1-\lambda )\frac{{q_{i}}^{T}k_{i}}{\tau }+\exp(\lambda \frac{{q_{i}}^{T}k_{\mathrm{ic}}}{\tau })}{\sum_{j=1}^{M}\exp(\frac{{q_{i}}^{T}{k_{j}}^{-}}{\tau })}$$

Where $q_{i}$ is the representation of the encoder embedding the original sample $x_{i}$ into a low-dimensional vector space, and $k_{i}$ is the representation of the dynamic encoder embedding the original sample $x_{i}$ into a compact vector space.$k_{\mathrm{ic}}$ is the representation of the dynamic encoder embedding the original sample $x_{i}$ neighbouring samples into a low-dimensional vector space. ${k_{j}}^{-}$ is the normalized cellular representation of the set of negative samples. λ is a hyperparameter that determines the weights between the pairs of positive samples. weights. M is the number of negative samples.

In order to facilitate the model to learn sparser feature representations, the $L_{1}$ regularisation term is introduced and the final loss function in comparison learning is.


(9) $$L_{i}={L_{i}}^{\mathrm{mixed}}+\alpha {\left|q_{i}\right|}_{1}$$

Where α is the hyperparameter used to balance the mixing InfoNCE loss and the $L_{1}$ regularisation term.

In this way, the hybrid InfoNCE loss function takes into account not only the original samples and their enhanced versions (positive samples), but also the neighbours of the samples (potentially positive samples), thus generating a cellular representation that is more conducive to clustering.

### 2.5 Self-knowledge distillation

Knowledge distillation follows the Teacher-Student framework, a method that entails transferring knowledge from a large, sophisticated, and high-performing teacher network to a more compact student network, enabling the student model to minimize computational demands while preserving strong performance. Specifically, the output of one pre-trained model (teacher model) is leveraged to assist in the training of another model (student model) (Yang *et al.* 2023). Self-knowledge distillation is a unique variant of knowledge distillation where the student and teacher models are the same. This is typically accomplished by applying various strategies during different phases of training, such as using an earlier iteration of the model to mentor a more advanced version (Yang *et al.* 2023).

In this research, the teacher model was constructed using the encoder framework from the contrastive learning model during the pre-training phase, which had captured valuable representations of the data. The student model employs the same framework as the teacher model but is trained after the pre-training phase, with the goal of learning to more effectively differentiate between data points, particularly in situations where clustering might be unclear or inaccurate during the pre-training stage.

In terms of implementation, after acquiring a compact, lower-dimensional portrayal of the data through the pre-training phase, initial clustering is performed using Leiden’s method to assign cluster labels to each cell, which are defined as pseudo-labels and are used to prepare for subsequent training and visualisation, such as UMAP or t-SNE. To prevent potential ambiguities or errors in clustering during the pre-training phase, cell anchors are identified to curate an elite subset of data for scKD to ensure data quality. In the specific implementation, the nearest neighbor set for each cell in the latent space is reconstructed using the latent feature representations obtained in the pre-training phase. Based on the generated pseudo-labels and the learned latent feature representations, the proximity of each cell within each cluster to its k nearest neighbors is assessed, and the mean proximity is calculated, which serves as the density metric for that point. The cell with a greater density metric within the cluster is considered the exemplar cell for that cluster and is designated as the anchor point. Subsequently, the top 40% of cells with the highest density in each cluster were identified as anchor cells. These cells were labeled as “low-density,” while the remaining cells were labeled as “high-density.” The low-density dataset was utilized as the training set, and the high-density dataset was employed as the test set, thereby generating a refined subset of high-quality data for model training and evaluation. The density metrics are defined as follows:


(10) $${\mathrm{Dens}}_{i}=\frac{1}{k}\sum_{j=1}^{k}\frac{{Z_{i}}^{T}Z_{j}}{{\left||Z_{i}|\right|}_{2}{\left||Z_{j}|\right|}_{2}}$$

Where the density of cell i is denoted by ${\mathrm{Dens}}_{i}$ and the embedding vectors of cell i and its particular neighbour cell j are denoted by $Z_{i}$ and $Z_{j}$, respectively.

To optimize the teacher model, the pre-trained encoder was reloaded, and a curated subset of high-quality data was employed to train the classification layer. The cross-entropy loss function was utilized to guide the training process, and its definition is provided below:


(11) $$H(p,\sigma (Z))=-\sum_{i=1}^{N}\sum_{c=1}^{C}p_{\mathrm{ic}}l\mathrm{og}({\sigma (Z_{i})}_{c})$$

The output from the teacher model is transferred to the student model as soft labels via the knowledge distillation technique. In other words, the student model is trained under the supervision of the teacher model, thereby enhancing the clustering performance. This process ultimately yields more accurate and reliable clustering labels as the final output of the student model, which is also the output of the self-KD model, through a customized deep clustering algorithm (Yang *et al.* 2023). The distillation loss function for the student model is presented below:


(12) $$L_{\mathrm{KD}}=\alpha H(p,{q_{s}}^{\tau 1})+(1-\alpha )({\tau_{2}}^{2})\mathrm{KL}({q_{t}}^{\tau 2},{q_{s}}^{\tau 2})$$

Where $\tau_{1}$ and $\tau_{2}$ denote the temperature in the softmax function. The distillation loss function comprises cross-entropy for hard labeling and KL divergence loss for soft labeling, with $q_{t}$ and $q_{s}$ representing the soft predictions of the teacher and student models, respectively. The hyperparameter α is used to balance these two losses.

## 3 Results

To assess the clustering effectiveness of scKD on single-cell RNA sequencing (scRNA-seq) data, it was tested on real datasets in this study and benchmarked against six state-of-the-art methods: scAnCluster (Chen *et al.* 2020c), scNAME (Wan *et al.* 2022), scDeepCluster (Tian *et al.* 2019), scNovel (Zheng *et al.* 2024), scDMFK (Chen *et al.* 2020b), ScMUG (Liang and Du 2025), Scanpy (Wolf *et al.* 2018), and scZidesk  (Chen et al., 2020a). Details of these methods are listed in Supplementary Table 3 (The selection criteria for the comparison methods in Table 3 of the Supplementary materials are specifically detailed in Supplementary Note 2). When assessing the clustering performance, three commonly used metrics are employed to evaluate the correspondence between the generated clusters and the ground-truth labels: the Adjusted Rand Index (Sundqvist *et al.* 2023) (ARI), the Normalised Mutual Information (Mahmoudi and Jemielniak 2024) (NMI) and the Normalised AMI (Lazarenko and Bonald 2021) (Adjusted Mutual Information, AMI).AMI and NMI have values ranging from 0 to 1, while ARI ranges from -1 to 1. These three metrics quantify the similarity of labels between the two clustering results, with higher values indicating greater similarity (The specific definitions and formulas of ARI, NMI, and AMI indicators are in the supplementary material “Supplementary Note 1” module).

### 3.1 Benchmarking against real scRNA-seq data

In this section, the study conducts a comparison between the proposed method and six existing methods using six real datasets, including scAnCluster, scNAME scDeepCluster, scNovel, scDMFK, ScMUG, Scanpy and scZidesk. in order to keep the benchmark comparisons under fair experimental conditions, for all benchmark datasets, a standardised preprocessing procedures and each method used its default parameter settings. Datasets from different species organs, different cell types, different cell numbers and different sequencing platforms were covered in this study to enable a more comprehensive comparative assessment (see Section 2.1 for specific dataset information). These datasets were obtained using various sequencing technologies, such as 10x Genomic, CELseq2, Smart-seq2, and inDrop. This diversity aims to demonstrate the robustness of the proposed method across different single-cell sequencing platforms and to confirm its applicability and reliability in diverse single-cell sequencing contexts. The experimental outcomes are presented in Supplementary Table 4, which includes the ARI, NMI, and AMI scores for scKD, the method introduced in this study, are relatively better when juxtaposed with the other methods, and in all the six datasets, only scKD has an ARI value of more than 0.9, whereas the other methods yield certain values that are below 0.9. In order to interpret the clustering results intuitively, The 2D planar visualization of the Muraro dataset is depicted in Supplementary Figure 1(A) where the colours of the cells correspond to the true labels provided in the dataset. In this case, scKD exhibits highly compact internal clustering and significant spacing between clusters, indicating superior clustering performance. Supplementary Figures 1(B) and (C) show box-and-line plots of the ARI values in these six experiments with real datasets. The scatter plots presented in Supplementary Figure 2 reveal the ranking of ARI values of the seven methods on the six datasets versus the scatter size, while the colours represent the ARI values.

### 3.2 Ablation experiments

Although contrastive learning has been applied in some areas of single-cell RNA sequencing analysis, there remain several challenges in improving the efficacy of these techniques and their use in cellular clustering tasks (Lee *et al.* 2023). In this study, scKD employs a contrast-enhanced learning strategy and a self-knowledge distillation model, with the goal of enhancing the effectiveness of contrastive learning techniques. To assess and demonstrate the efficacy of the proposed strategy in scKD, this study conducted ablation studies and used six real scRNA-seq datasets. The experiments consisted of four types of ablation experiments: removing the comparison model, removing the MLP, removing both the comparison model and the MLP, and removing both the selfKD, the comparison model, and the MLP. All ablation experiments were conducted using identical parameter configurations. Supplementary Table 5 presents the ARI, AMI, and NMI values achieved when evaluating the four distinct ablation experiment types across six real single-cell RNA sequencing datasets.

Supplementary Figure 3 illustrates the clustering outcomes of the four types of ablation experiments on the Mammary_Gland dataset. The results indicate that for the majority of the datasets, the contrast enhancement model and the self-knowledge distillation model have a positive impact on the clustering performance. Thus, the findings from the ablation study are in line with the expectation that the contrast-enhanced model helps the network to take into account the similarities between cells and capture the discriminative representations, while the self-knowledge distillation strategy can provide the ability to self-correct for the confusing cell types. The line graph in Supplementary Figure 4 illustrates the ARI values obtained when these four different ablation experiment types were evaluated on six real scRNA-seq datasets.

### 3.3 Model scKD accurately identifies rare cell populations in an in-dataset labelling task

Although many unsupervised scRNA-seq data analysis techniques have proven effective in identifying major cell subpopulations in recent years, challenges remain in revealing minor or rare cell types. Studies have shown that these rare cell subtypes, including stem cells, circulating tumor cells, and other minor cellular groups, often play key roles in tissue development and disease processes (Fu *et al.* 2019, Xie *et al.* 2020). To evaluate scKD’s ability to identify rare cell subtypes, the Baron_Human dataset was chosen as a benchmark for this study. This dataset, sourced from the human pancreas, contains 8569 cells, encompassing four rare cell subtypes: macrophage (0.64%), mast (0.29%), epsilon (0.21%), schwann (0.15%), and T cells (0.08%). In addition, scAnCluster, scNAME, scDeepCluster, scNovel, scDMFK, and scZidesk methods were used for comparison. To quantitatively evaluate the performance of each method, this study employs the F1 score (Chowdhury 2010) as the evaluation metric. The F1 score is the harmonic mean of precision and recall, ranging from 0 to 1, with higher values indicating better model performance (The specific definitions and formulas of F1-score indicators are in the supplementary material “Supplementary Note 1” module).

The results of the experiment are presented in Supplementary Figure 5, where all methods perform effectively in identifying major cell populations, such as Acinar and Alpha cells, which are abundant in the dataset and are easily and accurately detected by the model. However, when identifying cells with a relatively small percentage of cell types such as Macrophage cells and Mast cells, scKD demonstrated a more stable and accurate performance, being able to maintain a high recognition rate in low-frequency cell populations. On the contrary, the other methods failed to identify most of these rare cell types, showing some limitations. These results highlight the strengths of the scKD method in detecting rare cell subtypes. By performing self-knowledge distillation after contrastive learning, the ability of the model to discriminate these minority groups of cells was increased, providing a more reliable solution for cell type identification in scRNA-seq datasets. The percentages of all cell types in the Baron_Human dataset are shown in Supplementary Figure 6.A more detailed discussion of the limitations of this in-dataset rare cell identification benchmark and potential methodological extensions of scKD is provided in Supplementary Note 3.

## 4 Conclusion

This study presents an innovative approach for analyzing single-cell RNA sequencing data, scKD, which is based on hybrid neighbourhood contrast learning and self-knowledge distillation. The scKD method is designed to address the challenges of high dimensionality, complexity, and noise in analyzing single-cell variability, especially in accurately identifying cell types. By fusing the hybrid contrast learning model and the self-knowledge distillation strategy, scKD enhances the clustering performance of gene expression data at the single-cell level and effectively detects predominant cell types. Additionally, through comprehensive experiments on several authentic datasets, scKD shows outstanding results in cell subpopulation detection, clustering consistency, and robustness, outperforming other current state-of-the-art algorithms. Notably, compared to the majority of commonly used deep learning-driven scRNA-seq data analysis algorithms, scKD excels in annotation capabilities for rare cell types. It is worth noting that, compared to most widely used deep learning-based scRNA-seq data analysis algorithms, scKD demonstrates superior performance in annotating rare cell types.

In addition, to quantitatively assess model complexity and computational cost, we compared the number of parameters (Params) and floating-point operations (FLOPs) of nine representative methods, including scKD (see Supplementary Table 6). Specifically, scKD has 2.092 M Params and 5.239 M FLOPs, which is higher than lightweight methods such as Scanpy (0.033 M Params, 3.277 M FLOPs) and scNovel (0.139 M Params, 0.139 M FLOPs), but lower than more complex models such as scMUG (16.549 M Params, 16.523 M FLOPs).These results indicate that scKD trades a moderate increase in computational overhead for improved clustering performance and rare cell type annotation capability, but its application to large-scale datasets still has certain limitations. On datasets containing tens of thousands of cells, the relatively large number of parameters and FLOPs leads to increased runtime and memory consumption compared with lightweight baseline methods (see Supplementary Table 6), which may reduce its practicality in resource-constrained settings. To alleviate this issue, several improvements can be considered, such as introducing more parameter‑efficient network architectures and distilling scKD into a smaller student model to reduce model size and computational cost. In future work, we plan to systematically implement and evaluate these efficiency‑oriented strategies within the scKD framework, and further extend scKD to single‑cell multi‑omics data analysis to obtain a more comprehensive understanding of cellular heterogeneity. Previous studies have shown that single‑cell multi‑omics integration frameworks offer clear advantages in large‑scale data analysis. For example, MAESTRO (Ma *et al.* 2023) implements end‑to‑end processing of multi‑omics data, joint embedding and regulatory network analysis, and provides automatic cell type annotation and transcriptional regulator inference, thereby exhibiting favorable computational efficiency and scalability when handling large and complex single‑cell datasets. Inspired by these advances, we will consider incorporating similar multimodal joint modeling into scKD in the future, so as to further enhance its ability to resolve cellular states and regulatory mechanisms while maintaining computational efficiency.

## Supplementary Material

btag084_Supplementary_Data
