# Supplementary material for: A hybrid neighborhood enhanced contrastive learning and self-knowledge distillation method for scRNA-seq data clustering analysis
Source: Bioinformatics. 2026 Mar 29;42(3):btag084. doi: 10.1093/bioinformatics/btag084 (PMC13033185; doi:10.1093/bioinformatics/btag084)
Supplement: btag084_Supplementary_Data [file btag084_supplementary_data.zip › supplementary material.docx]

## A hybrid‑neighborhood enhanced contrastive learning and self‑knowledge distillation method for scRNA-seq data clustering analysis

## supplementary material

Supplementary Table 1 Biological and statistical information of the seven scRNA-seq datasets

| Datasets | Platform | Organ | Cell types | Cell Number | Reference |
| --- | --- | --- | --- | --- | --- |
| Baron_human | inDrop | Pancreas | 14 | 8596 | (Baron *et al.* 2016) |
| Mammary_Gland | 10x Genomic | Mammary Gland | 7 | 4481 | (Iram 2018) |
| Muraro | CEL-seq2 | Pancreas | 9 | 2122 | (Muraro *et al.* 2016) |
| Plasschaert | inDrop | Trachea | 8 | 6977 | (Plasschaert *et al.* 2018) |
| Human4 | inDrop | Pancreas | 14 | 4355 | (The Tabula Muris Consortium *et al.* 2018) |
| Limb Muscle | 10x Genomic | Mouse limb muscle | 6 | 11269 | (The Tabula Muris Consortium *et al.* 2018) |
| Quake_10x_Spleen | 10x Genomic | Spleen | 5 | 8552 | (The Tabula Muris Consortium *et al.* 2018) |

Supplementary Note 1：

**Evaluation Metrics for Clustering**

To assess the clustering performance of our model, we employed three widely used metrics: the Adjusted Rand Index (ARI) (Gao *et al.* 2021), Normalized Mutual Information (NMI) (Parulekar *et al.* 2023), and Adjusted Mutual Information (AMI) (Huang *et al.* 2022). Additionally, we included the F1-score for a comprehensive evaluation. These metrics quantify the agreement between the predicted cluster labels and the ground-truth labels, with higher values indicating better clustering consistency.

**Adjusted Rand Index (ARI) (Wang and Isola 2020):** ARI measures the similarity between two clusterings by considering all pairs of samples and counting pairs that are assigned in the same or different clusters in the predicted and true clusterings. It ranges from -1 to 1, where 1 indicates perfect agreement, 0 indicates random labeling, and negative values indicate less agreement than expected by chance. The formula is:

$$ARI=\frac{\sum_{i,j} (\begin{matrix} n_{ij} \\ 2 \end{matrix})-[\sum_{i} (\begin{matrix} a_{i} \\ 2 \end{matrix})\sum_{j} (\begin{matrix} b_{j} \\ 2 \end{matrix})]/(\begin{matrix} n \\ 2 \end{matrix})}{\frac{1}{2}[\sum_{i} (\begin{matrix} a_{i} \\ 2 \end{matrix})+\sum_{j} (\begin{matrix} b_{j} \\ 2 \end{matrix})]-[\sum_{i} (\begin{matrix} a_{i} \\ 2 \end{matrix})\sum_{j} (\begin{matrix} b_{j} \\ 2 \end{matrix})]/(\begin{matrix} n \\ 2 \end{matrix})}$$

Here, $n_{ij}$is the number of samples in both cluster i (predicted) and j (true), $a_{i}$ and $b_{j}$ are the sizes of the predicted and true clusters, and n is the total number of samples.

**Normalized Mutual Information (NMI) (Huang *et al.* 2022):** NMI evaluates the mutual dependence between the predicted and true labels, normalized to range from 0 to 1 (where 1 means perfect correlation). It is defined as:

$$NMI(E^{'},E)=\frac{2\cdot MI(E^{'},E)}{H(E^{'})+H(E)}$$

Where $MI(E^{'},E)$ is the mutual information between the predicted labels $E^{'}$ and true labels E, and $H(\cdot)$ is the entropy.

**Adjusted Mutual Information (AMI) (Wang *et al.* 2023):** AMI is an adjustment of mutual information that accounts for chance, similar to ARI. It also ranges from 0 to 1 and is particularly useful for imbalanced clusters.

$$AMI(U,V)=\frac{MI(U,V)-\mathbb{E[}MI(U,V)]}{max(H(U),H(V))-\mathbb{E[}MI(U,V)]}$$

$$MI(U,V)=\sum_{i=1}^{|U|} \sum_{j=1}^{|V|} P(i,j)\log(\frac{P(i,j)}{P(i)P(j)})$$

$$H(U)=-\sum_{i=1}^{|U|} P(i)\log P(i)$$

Where $MI(U,V)$ Mutual Information，$P(i,j)$ is the joint probability of a sample being in predicted cluster i and true cluster j, and $P(i)$, $P(j)$ are marginal probabilities. $H(U)$, $H(V)$ are Entropies of the clusterings.

**F1-Score (Lee *et al.* 2023):** This is the harmonic mean of precision and recall, ranging from 0 to 1, and is computed for each cluster before macro-averaging. It is useful for evaluating per-class performance in imbalanced datasets:

$$F1=2\cdot\frac{precision\cdot recall}{precision+recall}$$

Based on the normalised gene expression data, the top 1000 highly variable genes were selected for subsequent analysis. The experimental results are shown in Table 2 of the Supplementary Material.

Supplementary Table 2 ARI, NMI, and AMI values of scKD on six scRNA-seq datasets

|  | Metrics | scKD |
| --- | --- | --- |
| Mammar Gland | ARI | 0.9071 |
|  | NMI | 0.8812 |
|  | AMI | 0.8809 |
| Muraro | ARI | 0.7758 |
|  | NMI | 0.7926 |
|  | AMI | 0.7912 |
| Plasschaert | ARI | 0.9131 |
|  | NMI | 0.8423 |
|  | AMI | 0.8421 |
| Human4 | ARI | 0.8494 |
|  | NMI | 0.8231 |
|  | AMI | 0.8207 |
| Limb Muscle | ARI | 0.908 |
|  | NMI | 0.8912 |
|  | AMI | 0.891 |
| Quake 10x Spleen | ARI | 0.9271 |
|  | NMI | 0.8752 |
|  | AMI | 0.8744 |

Supplementary Table 3 Implement of other competitive methods

| Method | Programming  Language | Download URL | Reference |
| --- | --- | --- | --- |
| scAnCluster | Python | https://github.com/xuebaliang/scAnCluster | (Chen *et al.* 2020) |
| scNAME | Python | https://github.com/aster-ww/ scNAME | (Wan *et al.* 2022) |
| scDeepCluster | Python | https://  github.com/ttgump/scDeepCluster | (Tian *et al.* 2019) |
| scNovel | Python | https:// github.com/chuanyang-Zheng/scNovel | (Zheng *et al.* 2024) |
| scDMFK | Python | https://github.com/xuebaliang/scDMFK. | (Chen *et al.* 2020) |
| scZidesk | Python | :https://github.com/xuebaliang/  scziDesk. | (Chen *et al.* 2020) |
| ScMUG | Python | https://github.com/degiminnal/scMUG | (Liang and Du 2025) |
| Scanpy | Python | https://github.com/theislab/Scanpy | (Wolf *et al.* 2018) |

Supplementary Note 2：

To ensure a fair and comprehensive evaluation, the comparative methods listed in Supplementary Table 3 were selected based on three key criteria:

(1) representativeness: these methods are widely recognized or frequently cited in the field of single-cell transcriptomics, reflecting the current state-of-the-art in scRNA-seq clustering and representation learning;

1. methodological diversity: they cover different categories of algorithms, including deep learning-based (e.g., scAnCluster, scDeepCluster, scDMFK, scZidesk, scNAME, ScMUG, scNovel) and traditional framework-based approaches (e.g., Scanpy), thus enabling a balanced comparison across paradigms;
2. availability and reproducibility: all selected methods are open-source implementations with publicly available code, which ensures experimental reproducibility and objective performance comparison.
3. recency and up-to-dateness: we additionally prioritised methods that represent relatively recent advances in scRNA-seq analysis (mostly published within the past few years). This ensures that the comparison is performed against up to date architectures and training strategies (e.g. deep generative models, contrastive learning, and novel clustering losses), rather than only earlier baseline approaches. By doing so, the benchmarking better reflects the current development frontier of the field and provides a more stringent and informative reference for evaluating the practical advantages of scKD.

These selection principles allow the benchmarking to comprehensively reflect the strengths and weaknesses of various representative scRNA-seq analysis strategies and to rigorously validate the performance advantages of the proposed scKD model.

Supplementary Table 4 ARI, NMI, and AMI values of the seven competing methods on the six scRNA-seq datasets

|  | Metrics | ScAnCluster | ScNAME | ScDeepCluster | ScNovel | ScZidesk | ScDMFK | ScMUG | Scanpy | scKD |
| --- | --- | --- | --- | --- | --- | --- | --- | --- | --- | --- |
| Mammar Gland | ARI | 0.697 | 0.9008 | 0.6467 | 0.9103 | 0.6452 | 0.3242 | 0.4431 | 0.5865 | **0.9199** |
|  | NMI | 0.7955 | 0.8813 | 0.8186 | 0.8888 | 0.7966 | 0.393 | 0.6553 | 0.8106 | **0.8994** |
|  | AMI | 0.7949 | 0.881 | 0.818 | 0.8879 | 0.796 | 0.392 | 0.6545 | 0.8097 | **0.8992** |
| Muraro | ARI | 0.822 | 0.8826 | 0.5816 | 0.9258 | 0.3517 | 0.3276 | 0.4714 | 0.473 | **0.9312** |
|  | NMI | 0.8318 | 0.8365 | 0.7771 | 0.881 | 0.5914 | 0.5007 | 0.6157 | 0.7341 | **0.881** |
|  | AMI | 0.8291 | 0.835 | 0.7752 | 0.8785 | 0.5876 | 0.4959 | 0.6126 | 0.7306 | **0.8802** |
| Plasschaert | ARI | 0.6239 | 0.4923 | 0.329 | 0.8873 | 0.876 | 0.1411 | 0.2945 | 0.3111 | **0.9177** |
|  | NMI | 0.6976 | 0.6393 | 0.5546 | 0.8264 | 0.8322 | 0.2863 | 0.5212 | 0.5816 | **0.8446** |
|  | AMI | 0.6964 | 0.6384 | 0.5532 | 0.825 | 0.8317 | 0.2845 | 0.5201 | 0.5794 | **0.8444** |
| Human4 | ARI | 0.8127 | 0.6523 | 0.1596 | 0.91 | 0.3001 | 0.2967 | 0.3987 | 0.3538 | **0.9117** |
|  | NMI | 0.7554 | 0.6366 | 0.4231 | 0.8711 | 0.4526 | 0.3387 | 0.6586 | 0.674 | **0.8976** |
|  | AMI | 0.7534 | 0.6351 | 0.419 | 0.8694 | 0.4504 | 0.336 | 0.6575 | 0.6712 | **0.8964** |
| Limb Muscle | ARI | 0.5266 | 0.5531 | 0.1587 | 0.8326 | 0.694 | 0.7802 | 0.2973 | 0.1242 | **0.9752** |
|  | NMI | 0.6963 | 0.7195 | 0.5141 | 0.7761 | 0.6721 | 0.4801 | 0.5513 | 0.4865 | **0.9548** |
|  | AMI | 0.6958 | 0.7194 | 0.5133 | 0.7757 | 0.6718 | 0.4797 | 0.551 | 0.4853 | **0.9547** |
| Quake 10x Spleen | ARI | 0.8639 | 0.9214 | 0.1701 | 0.8622 | 0.9146 | 0.2476 | 0.001 | 0.2125 | **0.9218** |
|  | NMI | 0.8027 | 0.8604 | 0.433 | 0.7578 | 0.8418 | 0.2609 | 0.0014 | 0.478 | **0.867** |
|  | AMI | 0.8023 | 0.8603 | 0.4319 | 0.7572 | 0.8416 | 0.2599 | 0.0007 | 0.4766 | **0.866** |

Supplementary Table 5 ARI, NMI, and AMI values for the 4 types of ablation studies on the 6 scRNA-seq data sets

|  | Metrics | Original model | Without comparison model | Without MLP | Without comparison model and the MLP | Without comparison model,the MLP and KD |
| --- | --- | --- | --- | --- | --- | --- |
| Mammary Gland | ARI | **0.9199** | 0.687 | 0.448 | 0.6933 | 0.503 |
|  | NMI | **0.8994** | 0.7282 | 0.6446 | 0.7282 | 0.6137 |
|  | AMI | **0.8992** | 0.7277 | 0.644 | 0.7276 | 0.6131 |
| Muraro | ARI | **0.9312** | 0.7785 | 0.8448 | 0.7259 | 0.8455 |
|  | NMI | **0.881** | 0.7991 | 0.8371 | 0.7479 | 0.8287 |
|  | AMI | **0.8802** | 0.7977 | 0.8359 | 0.7462 | 0.8275 |
| Plasschaert | ARI | **0.9177** | 0.7578 | 0.9145 | 0.7655 | 0.7662 |
|  | NMI | **0.8446** | 0.7025 | 0.8436 | 0.6941 | 0.6977 |
|  | AMI | **0.8444** | 0.702 | 0.8442 | 0.6938 | 0.6974 |
| Human4 | ARI | **0.9117** | 0.8808 | 0.1112 | 0.7301 | 0.1222 |
|  | NMI | **0.8976** | 0.8337 | 0.2198 | 0.7357 | 0.2591 |
|  | AMI | **0.8964** | 0.8319 | 0.2137 | 0.7335 | 0.2531 |
| Limb Muscle | ARI | **0.9752** | 0.7515 | 0.8577 | 0.7649 | 0.738 |
|  | NMI | **0.9548** | 0.7738 | 0.8652 | 0.7782 | 0.7827 |
|  | AMI | **0.9547** | 0.7734 | 0.865 | 0.7778 | 0.7825 |
| Quake 10x Spleen | ARI | **0.9218** | 0.7961 | 0.9138 | 0.798 | 0.7972 |
|  | NMI | **0.867** | 0.7199 | 0.8536 | 0.7141 | 0.7118 |
|  | AMI | **0.866** | 0.7197 | 0.8528 | 0.7139 | 0.7115 |

Supplementary Material Table 6 demonstrates the comparison of the nine models selected in this study in terms of both the number of parameters (Params, in millions, M) and the number of floating point operations (FLOPs, in millions, M). The purpose is to facilitate a visual comparison of the differences in resource consumption, complexity and computational efficiency of the different models.

Supplementary Table 6 Params vs. FLOPs for 9 models

| Method | Params(M) | FLOPs(M) |
| --- | --- | --- |
| scKD | 2.092 | 5.239 |
| scNovel | 0.139 | 0.139 |
| scName | 4.555 | 4.549 |
| scDeepCluster | 5.173 | 6.455 |
| scDMFK | 0.196 | 0.194 |
| scAnCluster | 0.276 | 0.276 |
| scZidesk | 0.196 | 3.111 |
| scMUG | 16.549 | 16.523 |
| Scanpy | 0.033 | 3.277 |

Supplementary Figure 1：A: Clustering visualisation of the nine scRNA-seq data analysis methods; B: ARI box plots of scKD on different datasets(n=5 per box plot); C: ARI box plots of the seven scRNA-seq data analysis methods(n=5 per box plot);


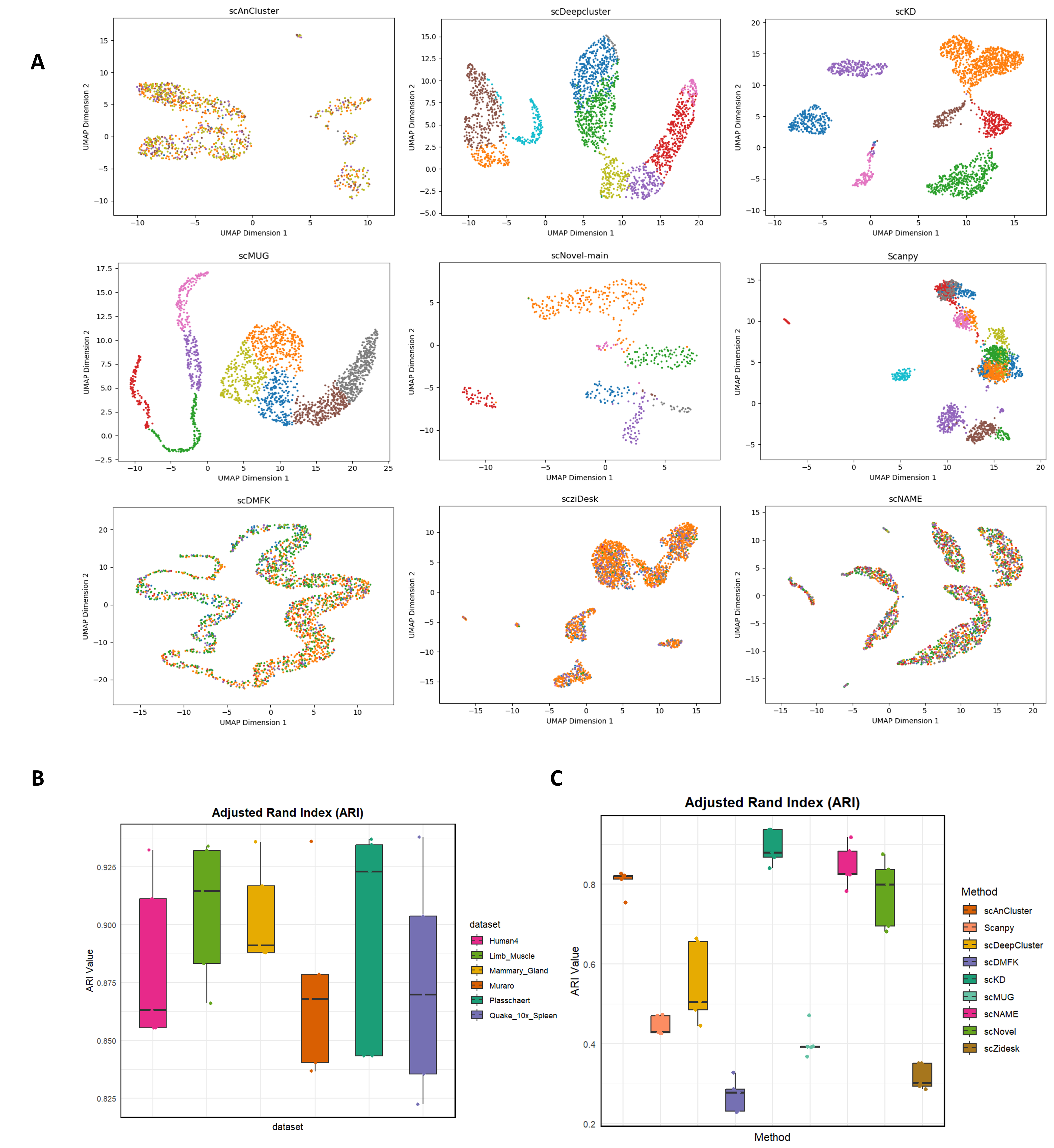


Supplementary Figure 2:ARI numerical dot plots of the seven scRNA-seq data analysis methods on the six datasets


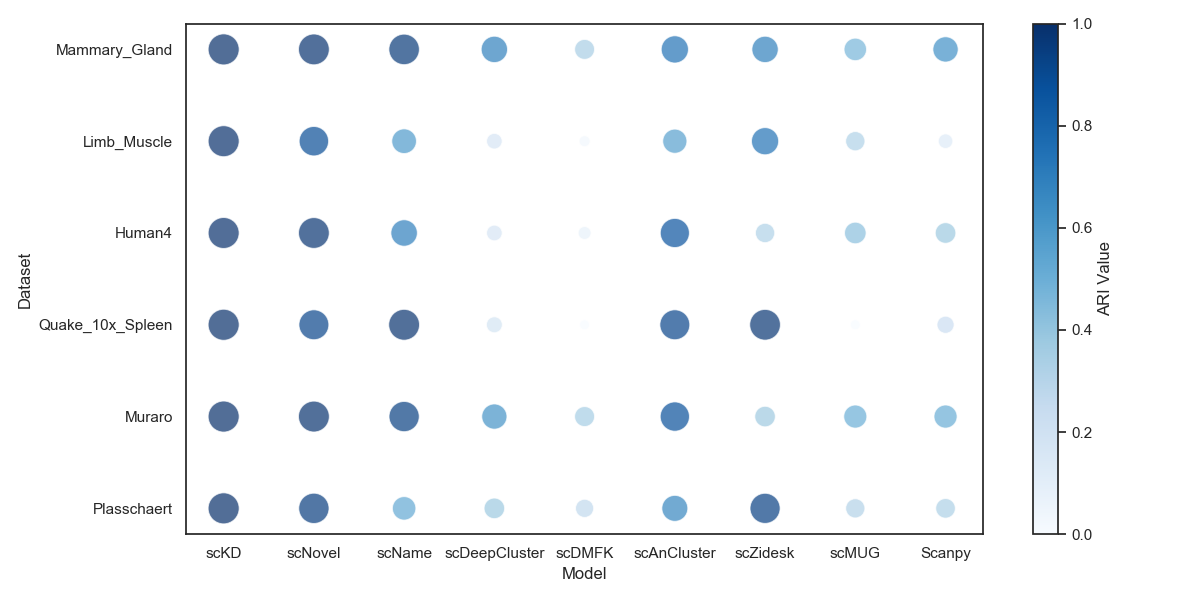


Supplementary Figure 3: Clustering visualisation of the 4 ablation experiments versus the original model on the Mammary Gland dataset;


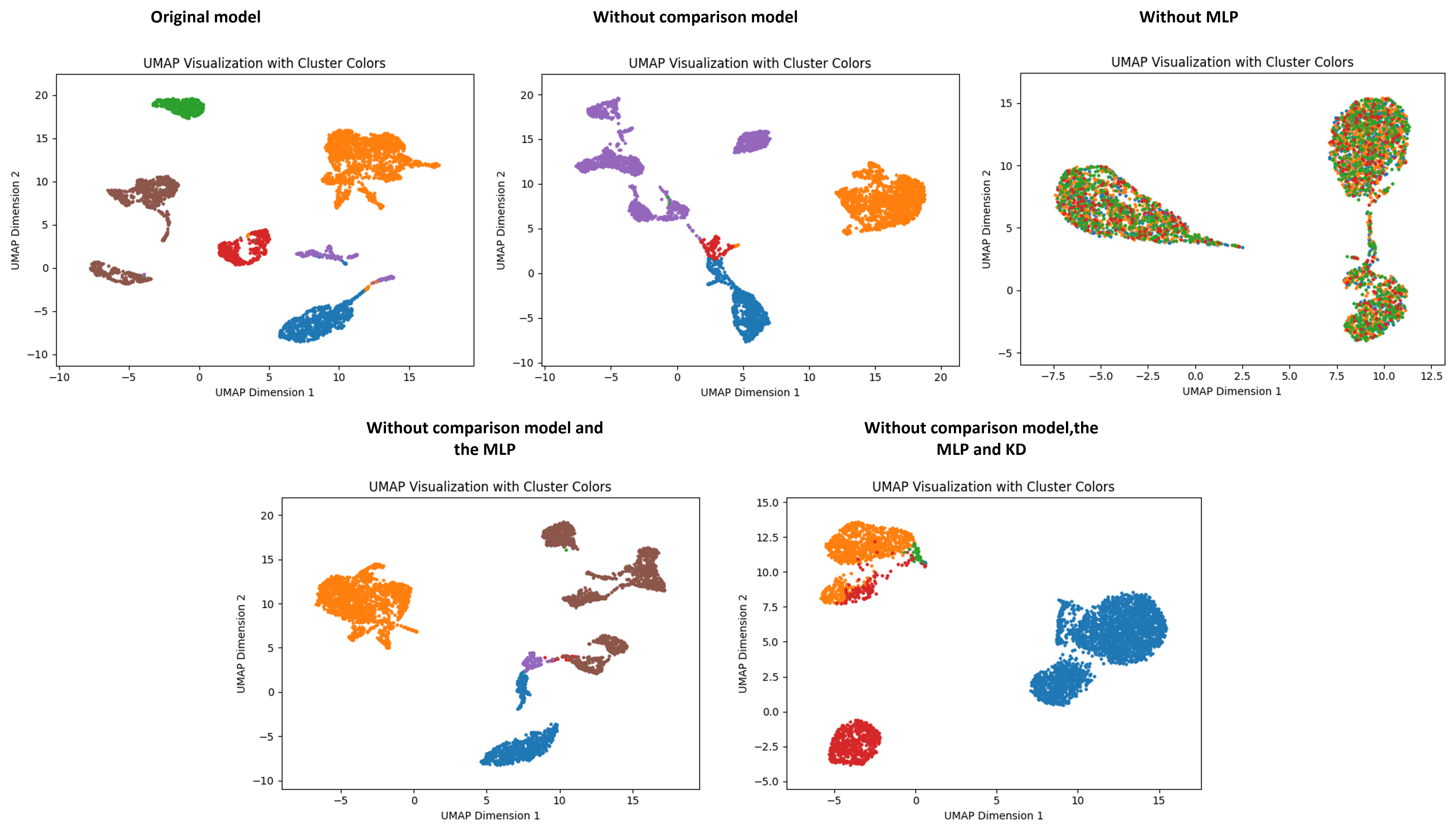


Supplementary Figure 4:Comparison of ARI scores of the 4 ablation experiments versus the original model on the 6 real scRNA-seq datasets in a line graph;


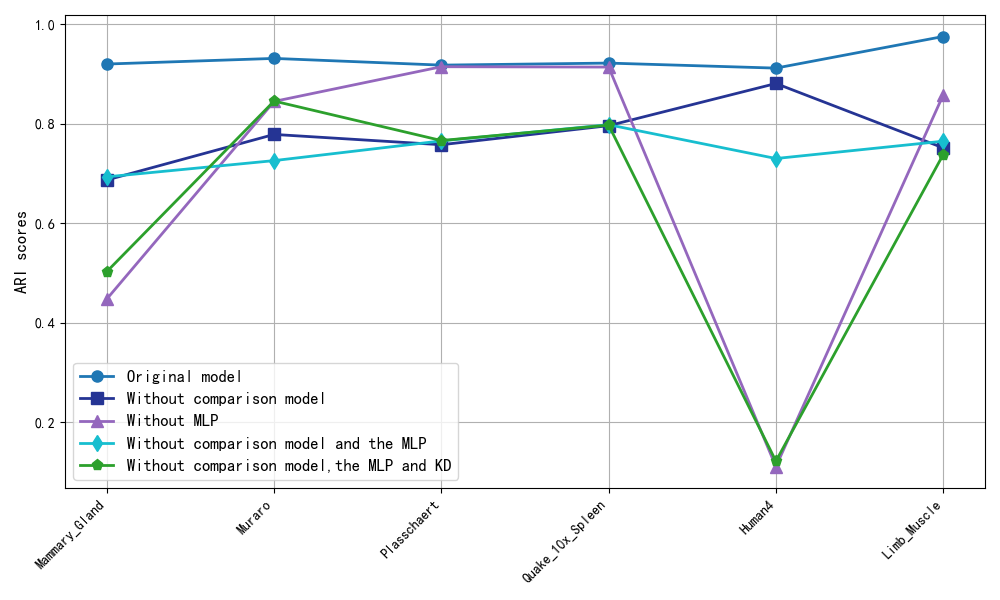


Supplementary Figure 5: Benchmarking the accuracy of identifying specific cell types on the Baron_Human dataset(n=5 per box plot), where the number to the right of each cell type name represents the number of cells of that cell type;


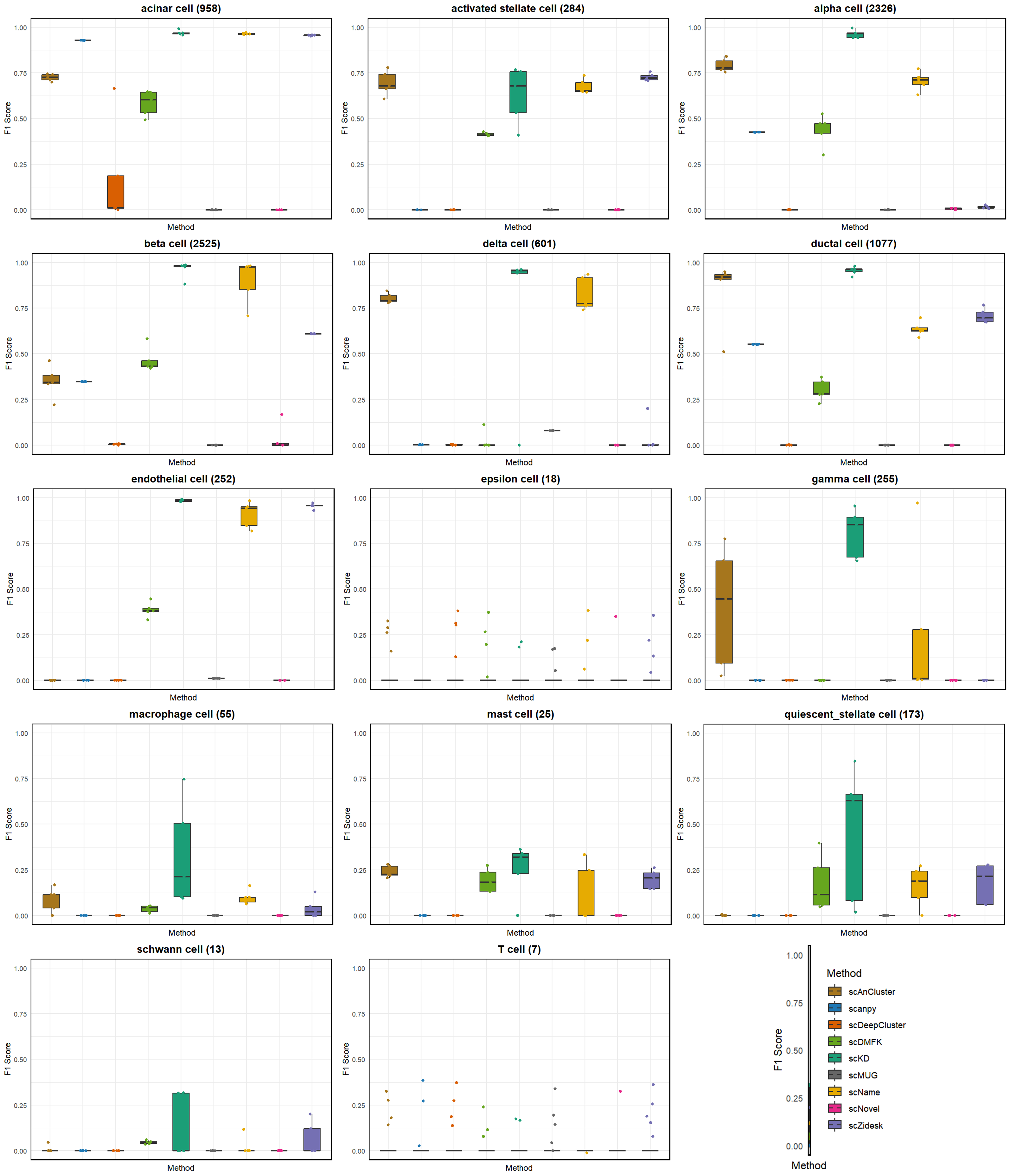


Supplementary Figure 6:Percentage of all cell types in the Baron_Human dataset;


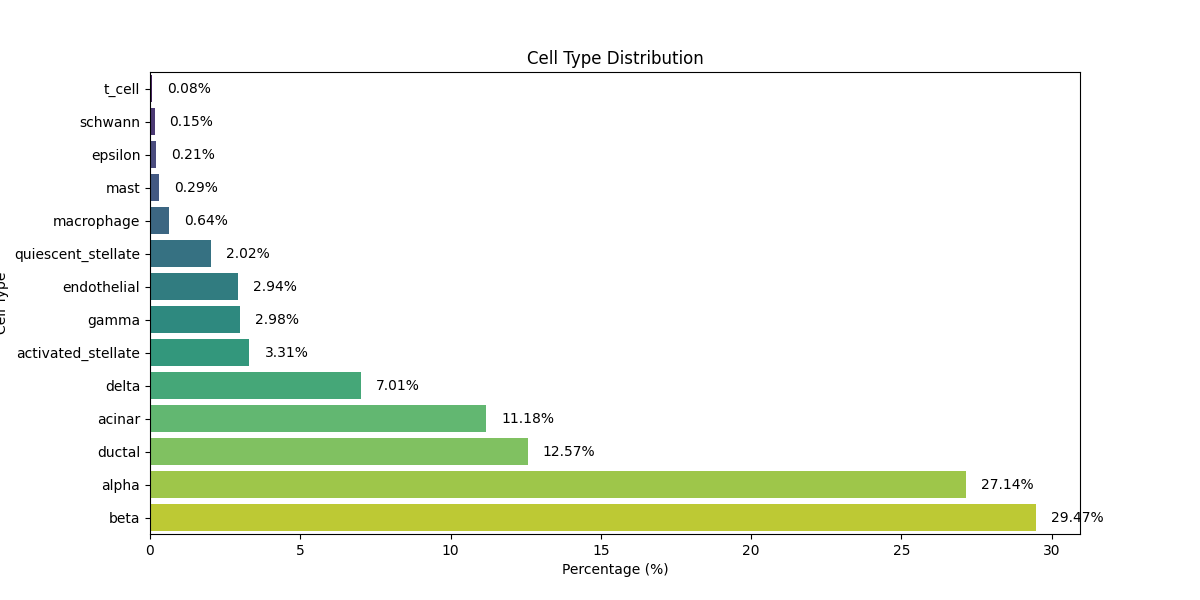


Supplementary Note 3：

Although the results in this section indicate that scKD can robustly identify rare cell populations, it should be noted that this evaluation setting is relatively favourable. The rare populations considered here still contain a moderate number of cells, exhibit clearly distinguishable transcriptomic signatures from other cell types in the same dataset, and are supported by complete reference labels. Therefore, while the practical advantage of scKD is consistent, it is not overwhelmingly large.

Moreover, this in-dataset evaluation does not fully cover several challenging yet practically relevant scenarios, such as: (i) “ultra-rare” populations consisting of only a handful of cells; (ii) rare cell states that are only weakly separated from major lineages or lie on continuous differentiation trajectories; and (iii) situations where reference labels are incomplete or partially noisy. In these more difficult cases, the performance gains brought by the hybrid contrastive learning module and the self-knowledge distillation strategy may be attenuated, and additional prior knowledge or targeted experimental validation would still be required to reliably define rare cell states.

To alleviate these issues, several potential extensions can be considered. First, one could explicitly incorporate “rarity-aware” or “uncertainty-aware” weighting into the contrastive learning objective, for example by up-weighting low-density regions or samples with high prediction entropy, so as to better preserve weakly separated or extremely rare cell states. Second, scKD could be integrated with dedicated trajectory inference or manifold learning tools in a two-stage pipeline: continuous differentiation processes are first characterised by these methods, and scKD is then applied to sharpen the separation of discrete subpopulations, thereby leveraging its clustering strengths while retaining the underlying continuum structure. Third, adaptive quality-control and feature-selection strategies tailored to low-coverage cells could be developed and coupled with the ZINB encoder to improve robustness on extremely sparse datasets.

Overall, under the current in-dataset benchmark for rare cell identification, the contribution of scKD is better viewed as an incremental improvement rather than a transformative breakthrough. At the same time, the above potential extensions suggest that, without changing the overall framework, scKD remains flexible and can be further optimised for more challenging and practically relevant rare-cell scenarios.

**References**

Baron M, Veres A, Wolock SL *et al.* A single-cell transcriptomic map of the human and mouse pancreas reveals inter-and intra-cell population structure. *Cell Syst* 2016;**3**:346–60. e4.

Chen L, Wang WN, Zhai YY *et al.* Deep soft K-means clustering with self-training for single-cell RNA sequence data. *NAR Genomics Bioinf* 2020;**2**:lqaa039.

Chen L, Wang WN, Zhai YY *et al.* Single-cell transcriptome data clustering via multinomial modeling and adaptive fuzzy k-means algorithm. *Front Genet* 2020;**11**:295.

Chen L, Zhai YY, He QY *et al.* Integrating deep supervised, self-supervised and unsupervised learning for single-cell RNA-seq clustering and annotation. *Genes* 2020;**11**:792.

Gao TY, Yao XC, Chen DQ. Simcse: Simple contrastive learning of sentence embeddings. In: *Proceedings of the 2021 conference on empirical methods in natural language processing*. *Online and Punta Cana, Dominican Republic* *2021*. p.6894–910. Online and Punta Cana, Dominican Republic: Association for Computational Linguistics, 2021.

Huang ZZ, Chen J, Zhang JP *et al.* Learning representation for clustering via prototype scattering and positive sampling. *IEEE Trans Pattern Anal Mach Intell* 2022;**45**:7509–24.

Iram T. Single-cell transcriptomics of 20 mouse organs creates a Tabula Muris. *Nature* 2018;**562**:367–72.

Lee J, Kim S, Hyun D *et al.* Deep single-cell RNA-seq data clustering with graph prototypical contrastive learning. *Bioinformatics* 2023;**39**:btad342.

Liang DM, Du PF. scMUG: Deep clustering analysis of single-cell RNA-seq data on multiple gene functional modules. *Brief Bioinform* 2025;**26**:bbaf138.

Muraro MJ, Dharmadhikari G, Grün D *et al.* A single-cell transcriptome atlas of the human pancreas. *Cell Syst* 2016;**3**:385–94. e3.

Parulekar A, Collins L, Shanmugam K *et al.* Infonce loss provably learns cluster-preserving representations. In: *The thirty sixth annual conference on learning theory*. *New York* *2023*. p.1914–61. New York: PMLR, 2023.

Plasschaert LW, Žilionis R, Choo-Wing R *et al.* A single-cell atlas of the airway epithelium reveals the CFTR-rich pulmonary ionocyte. *Nature* 2018;**560**:377–81.

The Tabula Muris Consortium, Overall coordination, Logistical coordination *et al.* Single-cell transcriptomics of 20 mouse organs creates a Tabula Muris. *Nature* 2018;**562**:367–72.

Tian T, Wan J, Song Q *et al.* Clustering single-cell RNA-seq data with a model-based deep learning approach. *Nat Mach Intell* 2019;**1**:191–8.

Wan H, Chen L, Deng MH. scNAME: Neighborhood contrastive clustering with ancillary mask estimation for scRNA-seq data. *Bioinformatics* 2022;**38**:1575–83.

Wang TZ, Isola P. Understanding contrastive representation learning through alignment and uniformity on the hypersphere. In: *International conference on machine learning*. *New York* *2020*. p.9929–39. New York: PMLR, 2020.

Wang ZY, Wang P, Palpanas T *et al.* Graph-and tree-based indexes for high-dimensional vector similarity search: Analyses, comparisons, and future directions. *IEEE Data Eng Bull* 2023;**47**:3–21.

Wolf FA, Angerer P, Theis FJ. SCANPY: Large-scale single-cell gene expression data analysis. *Genome Biol* 2018;**19**:15.

Zheng CY, Wang YX, Cheng YQ *et al.* scNovel: A scalable deep learning-based network for novel rare cell discovery in single-cell transcriptomics. *Brief Bioinform* 2024;**25**:bbae112.
