# Supplementary figures and images for: A hybrid neighborhood enhanced contrastive learning and self-knowledge distillation method for scRNA-seq data clustering analysis
Source: Bioinformatics. 2026 Mar 29;42(3):btag084. doi: 10.1093/bioinformatics/btag084 (PMC13033185; doi:10.1093/bioinformatics/btag084)

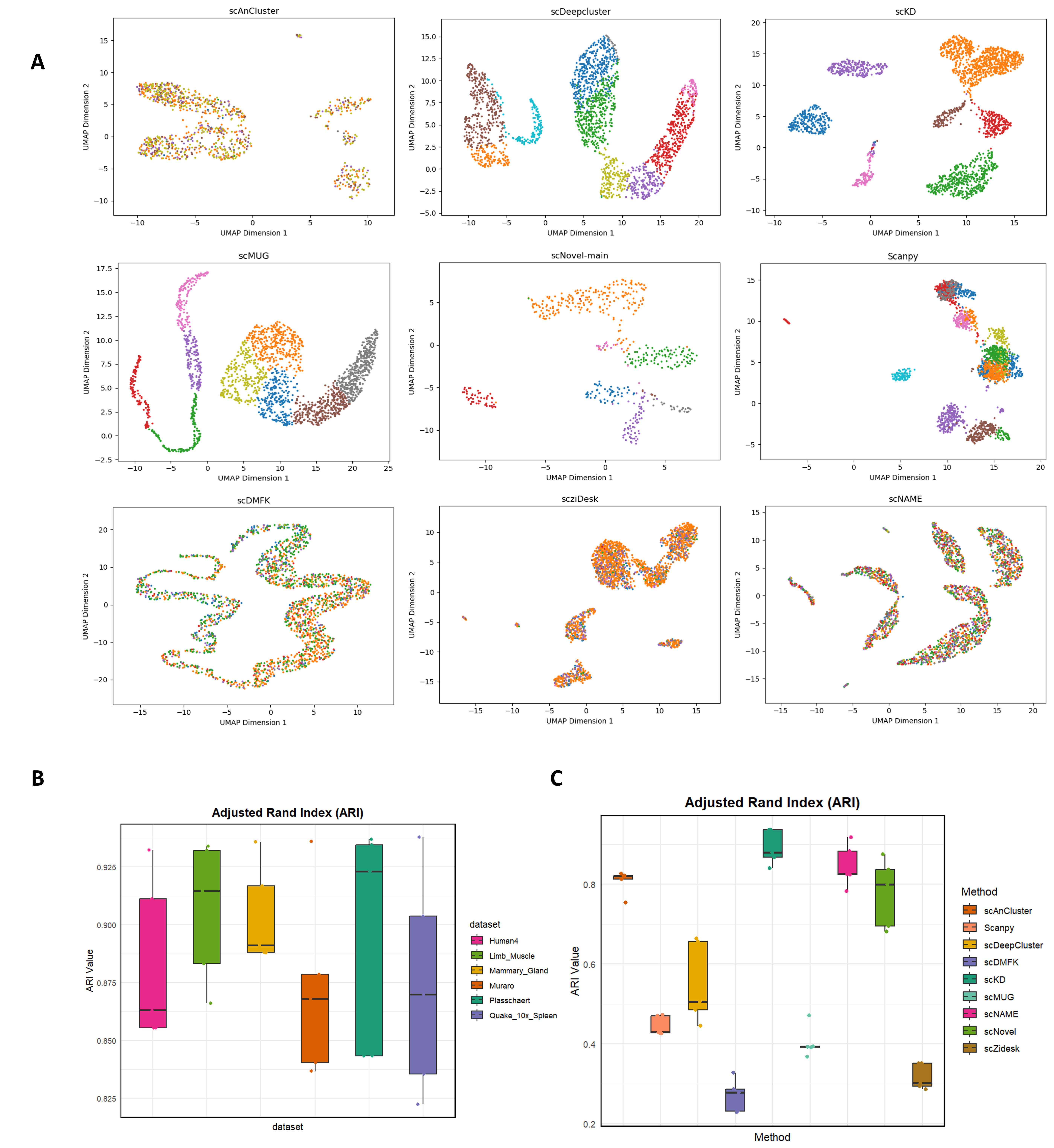

Supplement: btag084_Supplementary_Data [file btag084_supplementary_data.zip › Supplementary Figure 1.tif]

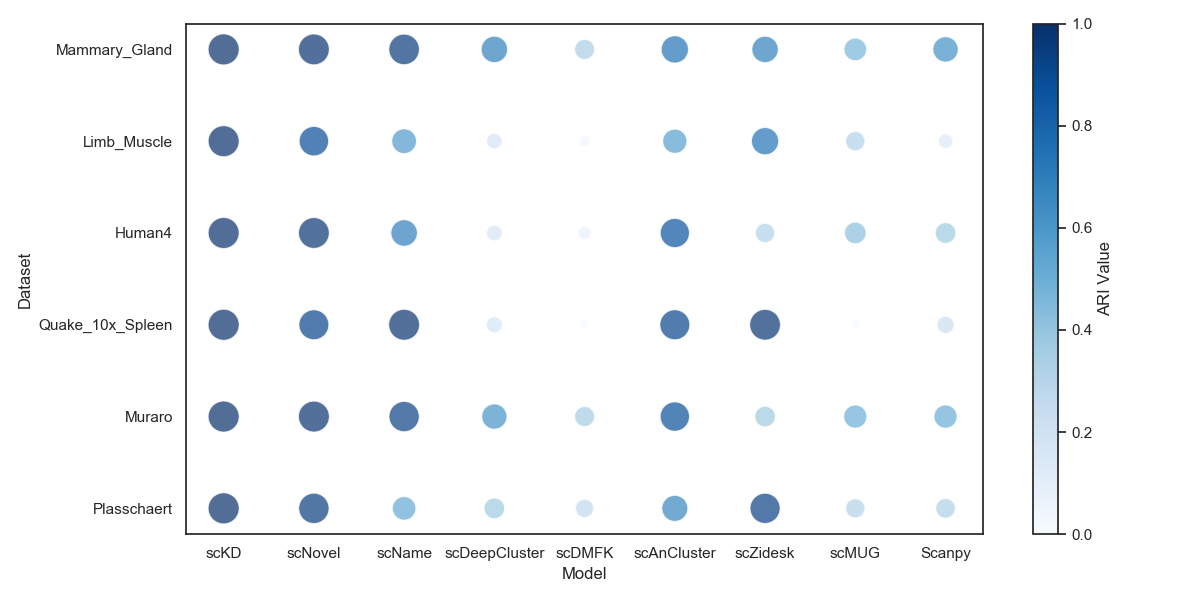

Supplement: btag084_Supplementary_Data [file btag084_supplementary_data.zip › Supplementary Figure 2.png]

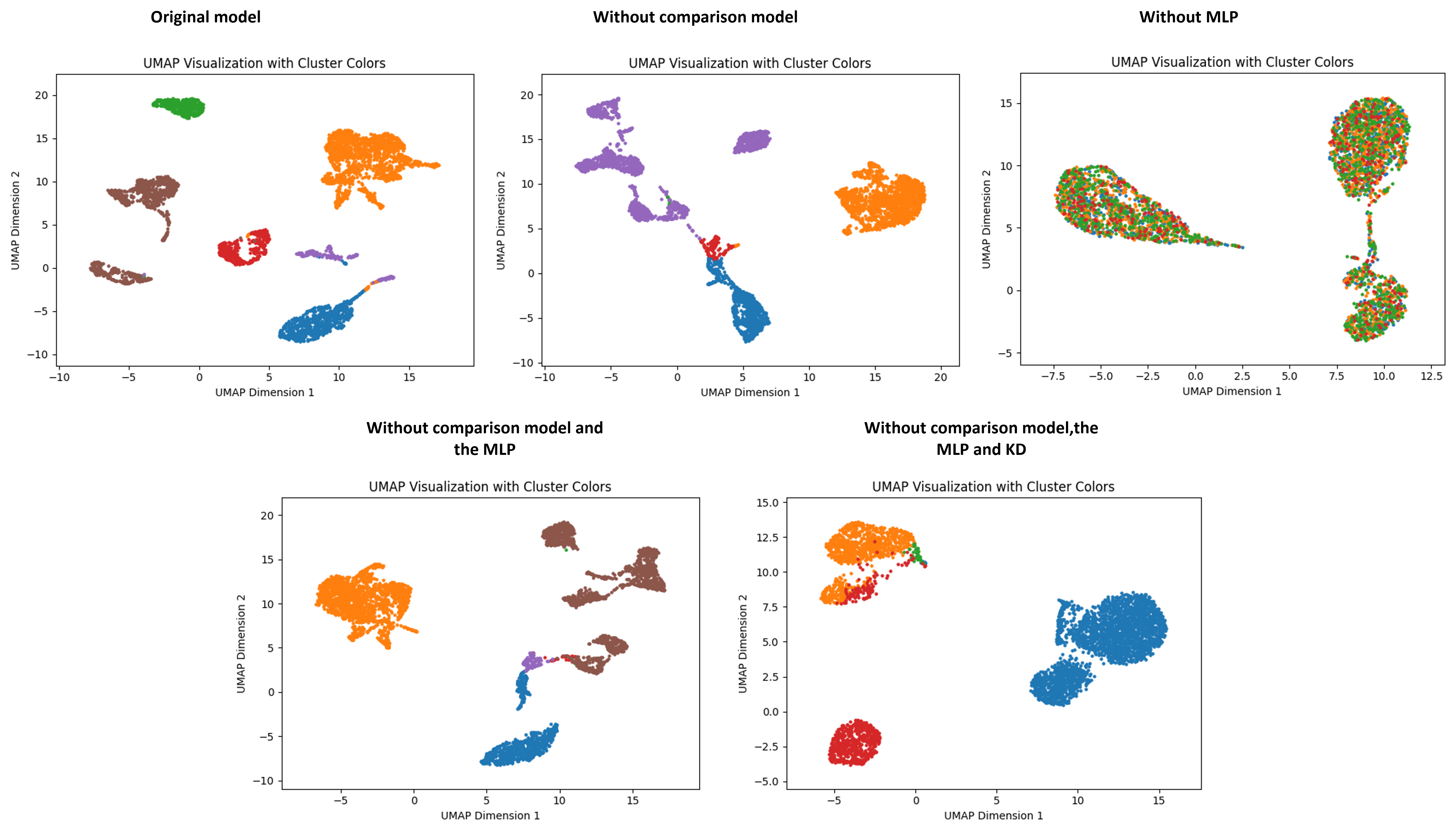

Supplement: btag084_Supplementary_Data [file btag084_supplementary_data.zip › Supplementary Figure 3.tif]

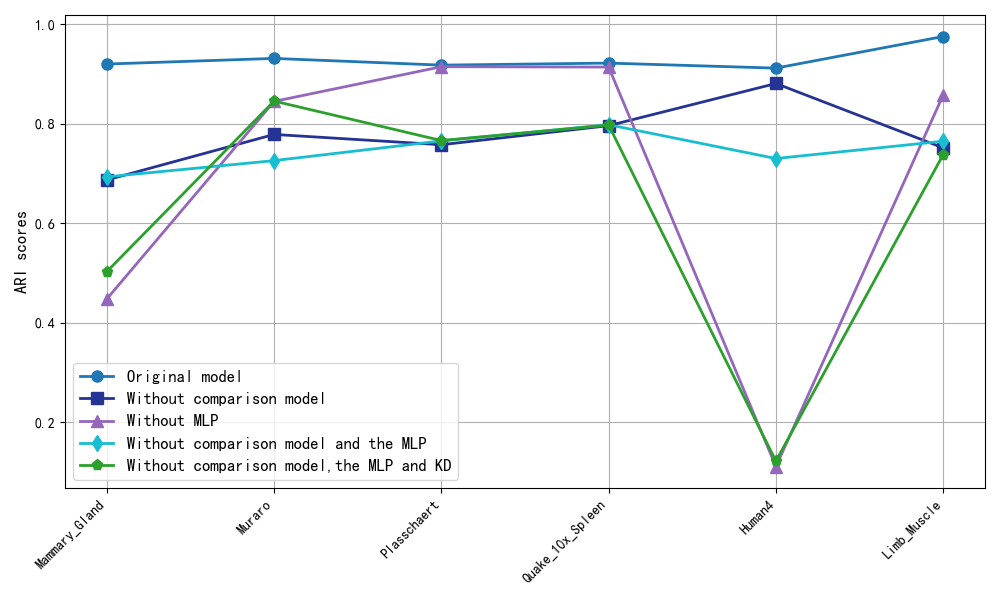

Supplement: btag084_Supplementary_Data [file btag084_supplementary_data.zip › Supplementary Figure 4.png]

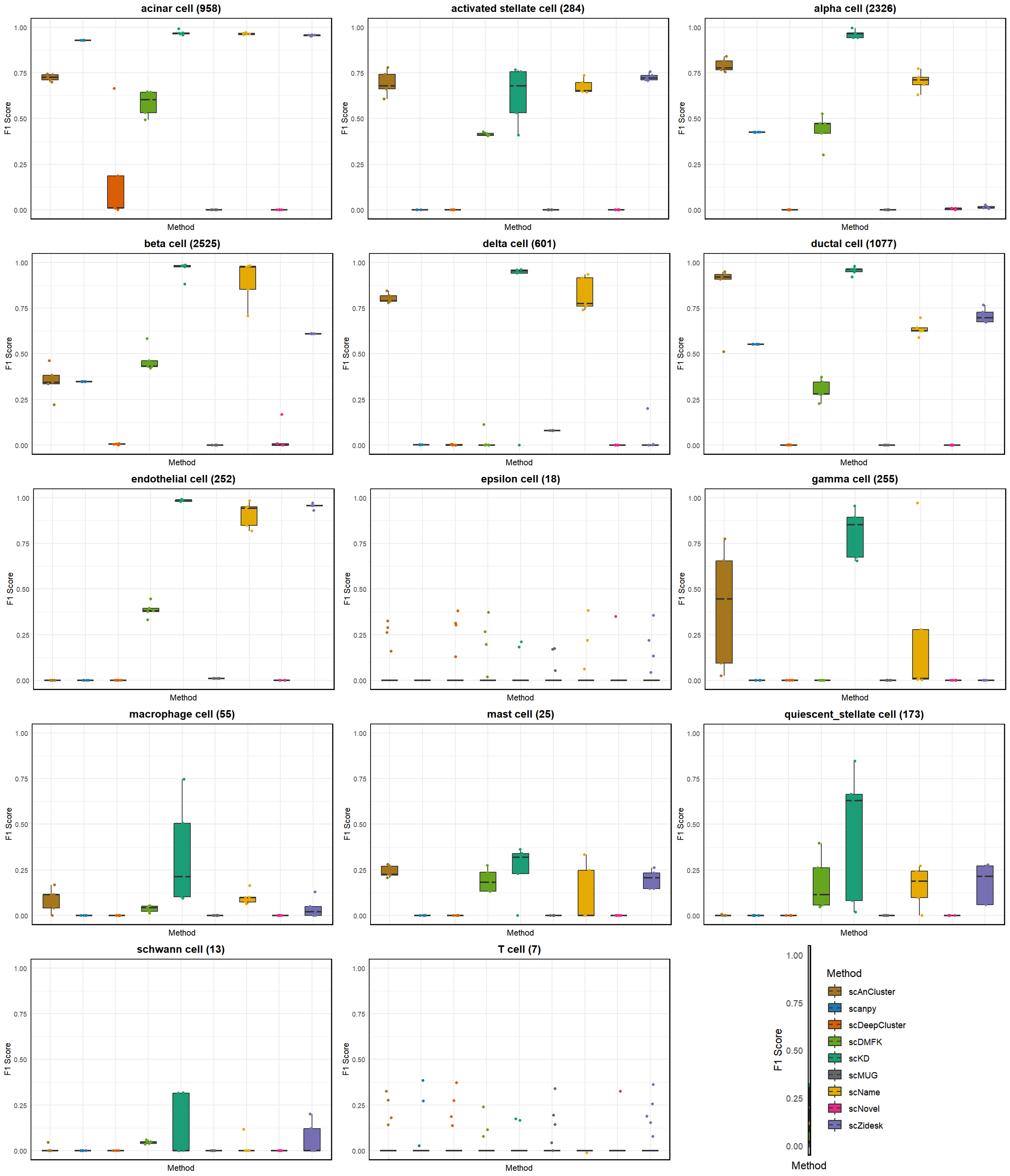

Supplement: btag084_Supplementary_Data [file btag084_supplementary_data.zip › Supplementary Figure 5.tif]

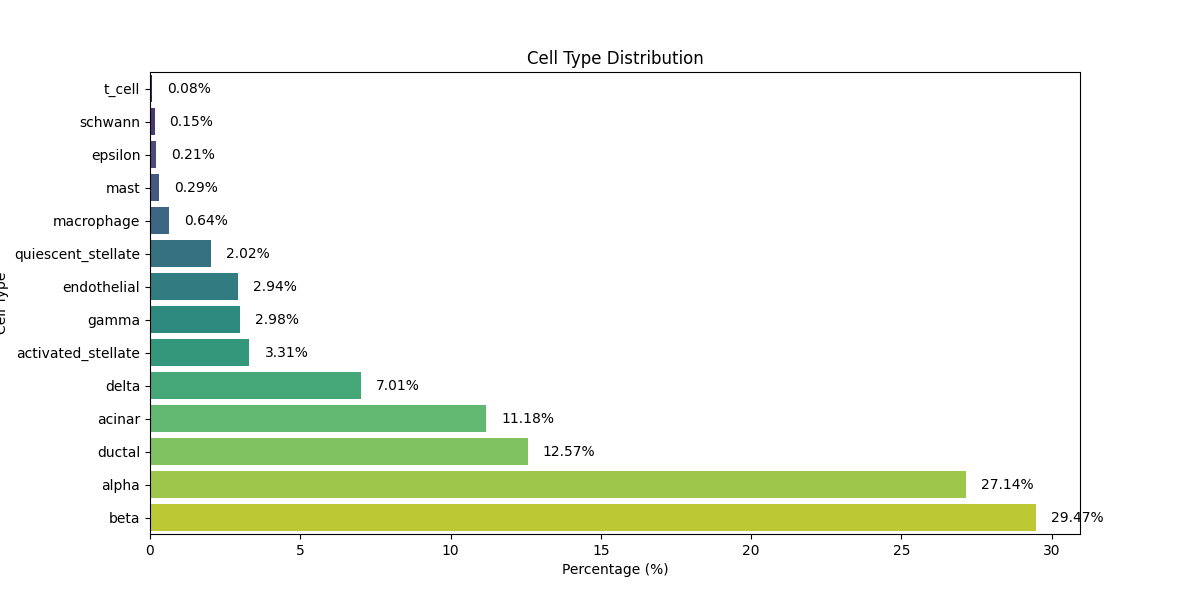

Supplement: btag084_Supplementary_Data [file btag084_supplementary_data.zip › Supplementary Figure 6.png]
